# Receptor tyrosine kinases (RTKs) in breast cancer: signaling, therapeutic implications and challenges

**DOI:** 10.1186/s12943-018-0797-x

**Published:** 2018-02-19

**Authors:** Ramesh Butti, Sumit Das, Vinoth Prasanna Gunasekaran, Amit Singh Yadav, Dhiraj Kumar, Gopal C. Kundu

**Affiliations:** 1 Laboratory of Tumor Biology, Angiogenesis and Nanomedicine Research, National Centre for Cell Science, SP Pune University Campus, Pune, 411007 India; 2 Department of Cancer Biology, The University of Texas MD Anderson Cancer Center, Houston, Texas 77054 USA

**Keywords:** Brest cancer, Tumor microenvironment, Angiogenesis, Cancer Stem cells, Tumor-stroma interaction, Metastasis, Anti-RTK therapy, Drug resistance, Lapatinib, Trastuzumab, Bevacizumab, Alternate pathway activation

## Abstract

Breast cancer is a multifactorial disease and driven by aberrant regulation of cell signaling pathways due to the acquisition of genetic and epigenetic changes. An array of growth factors and their receptors is involved in cancer development and metastasis. Receptor Tyrosine Kinases (RTKs) constitute a class of receptors that play important role in cancer progression. RTKs are cell surface receptors with specialized structural and biological features which respond to environmental cues by initiating appropriate signaling cascades in tumor cells. RTKs are known to regulate various downstream signaling pathways such as MAPK, PI3K/Akt and JAK/STAT. These pathways have a pivotal role in the regulation of cancer stemness, angiogenesis and metastasis. These pathways are also imperative for a reciprocal interaction of tumor and stromal cells. Multi-faceted role of RTKs renders them amenable to therapy in breast cancer. However, structural mutations, gene amplification and alternate pathway activation pose challenges to anti-RTK therapy.

## Background

Breast cancer is a major cause of morbidity and mortality among women population worldwide. The incidence of breast cancer differs considerably worldwide. It is expected to affect 0.2 million and would result in an estimated 41,070 deaths in 2017 in USA [1]. Breast cancer emerges as a consequence of dysregulation of different signaling pathways in mammary epithelial cells. Growth factors and chemokines activate various signaling cascades which cross-talk in tumor microenvironment leading to cancer progression. They bind to different families of receptors. Receptor Tyrosine Kinases (RTKs) comprise one such family. RTKs are single-pass transmembrane proteins, expressed on various cell types including the ones in the tumor microenvironment. Overexpression of various types of RTKs such as epidermal growth factor receptors (EGFRs), vascular endothelial growth factor receptors (VEGFRs), platelet-derived growth factor receptors (PDGFRs), insulin-like growth factor receptors (IGFRs), and fibroblast growth factor receptors (FGFRs) is found in different types of cancer including breast [2–4]. Elevated levels of RTKs are associated with increased breast cancer aggressiveness and decreased overall and disease-free survival [5]. Ligand binding leads to conformational changes in RTKs that result in activation of downstream signaling molecules. The important pathways that are known to be activated by RTKs include mitogen-activated protein kinase (MAPK), Janus kinase (JAK)/ signal transducer and activator of transcription (STAT) and phosphoinositide 3-kinase (PI3K)/Akt [6–10]. RTK-regulated pathways play key roles in various facets of cancer progression. RTK-activated signaling also induces cancer stem cell (CSC) phenotype that exhibit resistance to therapeutic regimens [6, 9]. Cancer progression is not only regulated by autonomous signaling networks but also context-dependent molecular signals received from tumor stroma. Tumor stroma consists of various types of non-cancerous cells such as fibroblasts, endothelial cells, macrophages and other immune cells [11]. RTK signaling-regulated interplay between the tumor and stromal cells contributes to tissue remodeling, stromal cell recruitment and activation. Survival of disseminated cancer cells in metastatic sites requires formation of the pre-metastatic niche by stromal cells. Stromal cells expressing RTKs are known to be recruited to metastatic sites and have been found to form pre-metastatic niche through the RTK-regulated signaling [8]. RTKs also regulate trans-differentiation of cancer cells to endothelial cells to form new blood vessels in a process known as vasculogenic mimicry [12, 13]. Since RTKs play important roles in different aspects of breast cancer progression, targeting RTKs might be useful in cancer treatment. Over the years, several RTK inhibitors have been screened and tested in clinical trials. Some of them such as lapatinib, trastuzumab and bevacizumab have been approved by Food and Drug Administration (FDA), USA for clinical management of breast cancer. Interestingly, RTK inhibitors revert conventional therapy-induced multidrug resistance and improve the disease-free survival in metastatic breast cancer patients [14]. Even though anti-RTK therapy shows clinical benefits in breast cancer patients, unfortunately, cancer cells develop *de novo* or acquired resistance that limits the success of RTK-targeted therapy [15]. In this review, we deal with EGFR, VEGFR, PDGFR and FGFR signaling in breast cancer progression, maintenance of cancer stem cell phenotype, tumor-stroma interaction and drug resistance. Moreover, this review also discusses the major challenges in targeting RTKs for the successful treatment of breast cancer.

### Structure and classification of RTKs

**Fig. 1:**
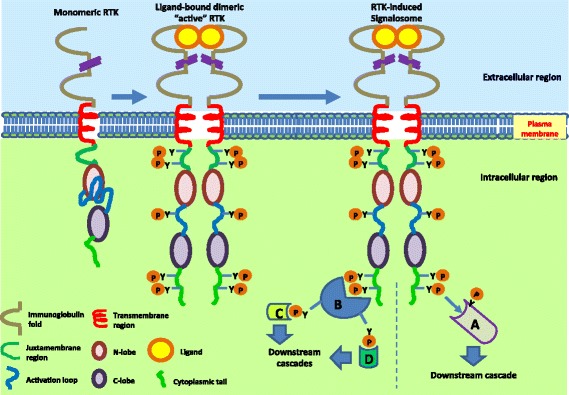
Structure of prototype of receptor tyrosine kinase and mechanism of activation. Receptor tyrosine kinases (RTKs) have the following structural segments from N- to C-terminal: immunoglobulin folds, transmembrane region, juxtamembrane region, N-lobe, activation loop, C-lobe and cytoplasmic tail. RTKs reside at the plasma membrane as a monomer. Ligand binding crosslinks receptor molecules and induces conformational changes that lead to receptor autophosphorylation and activation. Phosphorylated RTK either serves as a docking site for adaptor proteins (B) or may directly phosphorylate signaling molecules (A). Adaptor proteins or signaling molecules bind to phosphorylated receptor through Src homology 2 (SH2) or phosphotyrosine-binding (PTB) domain. Docked adaptor proteins further transduce signal by phosphorylating other downstream molecules (C, D)

Fifty eight different RTKs have been characterized in humans and they have been classified into 20 different subfamilies on the basis of structural features. Each RTK subfamily exhibits a prototype structural organization along with class-specific characteristics. A prototype RTK has an extracellular ligand-binding domain and intracellular tyrosine kinase domain separated by a transmembrane domain. The subfamilies of RTKs are (1) EGFR, (2) InsR, (3) PDGFR, (4) VEGFR, (5) FGFR, (6) PTK7/CCK4, (7) Trk, (8) Ror, (9) MuSK, (10) Met, (11) Axl, (12) Tie, (13) EphA/B, (14) Ret, (15) Ryk, (16) DDR1/2, (17) Ros, (18) LMR, (19) ALK and (20) SuRTK106/STYK1. The intracellular domain of RTKs has tyrosine kinase activity (tyrosine kinase domain; TKD). This tyrosine kinase domain can phosphorylate tyrosine residues in *cis* (within the same molecule) or in *trans* (residing on a different molecule) (Fig. 1). This consensus design of RTKs has been found to be conserved across evolution. Mutations in RTKs that result in structural abnormalities have been found to lead various disorders.

RTKs are activated by binding of soluble ligands. Some of the RTKs (DDR1, DDR2) are activated not by soluble ligands but by collagen fibers of the extracellular matrix [16]. Two compulsory events in RTK activation are ligand binding and receptor dimerization. Although the earlier idea was that cognate ligand binding ultimately results in the receptor dimerization, it has been found that few RTKs are oligomeric even in the absence of ligands [17]. EGFR is mostly present as a monomer whereas insulin receptor is present as a dimer on the cell membrane [18]. Nonetheless, receptor activation requires binding of ligand and consequent dimerization or oligomerization of the former in an active state. Different mechanisms for ligand binding-induced receptor dimerization have been explained for different classes of RTKs by different research groups. The mechanisms include two extremes where the dimer interface is formed entirely either by the ligand or the receptor molecules. The two other mechanisms include the participation of both ligand and receptor for the formation of the dimer interface and in another case participation of an accessory molecule. An example of the first mechanism is activation of nerve growth factor (NGF) receptor, TrkA where only two NGF molecules form the dimer interface and none of receptor extracellular domains make physical contact to the neighboring molecule [19, 20]. The ligands that activate members of the EGFR family do not themselves form dimers rather they bind two different domains of the same molecule and induce favorable conformational changes that lead to the formation of dimer interface by the receptor molecules [21]. Stem cell factor (SCF) binds to its receptor, KIT and induces receptor dimerization where the dimer interface is formed by both the ligand and receptor molecules [22]. In case of FGFR, heparin molecule stabilizes FGFR dimer configuration following ligand (fibroblast Growth factor (FGF)) binding [23].

In the absence of cognate ligands, the RTKs are held in an inactive state by autoinhibitory mechanisms. Two different autoinhibitory mechanisms have been described for different families of RTKs. The TKD of the RTKs contains three essential elements, N lobe, C lobe and activation loop [24]. In the activation loop-mediated autoinhibitory mechanism, the activation loop makes physical contact with the active site of TKD. A critical tyrosine residue in the activation loop is phosphorylated and the tyrosine kinase activity is autoinhibited in *cis* [25]. In the other mechanism, juxtamembrane sequences make extensive contact with the active site of the TKD and the latter is arrested in an autoinhibited inactive conformation [26–28]. Ligand binding induces favorable conformational changes that get rid of autoinhibitions following receptor dimerization. Activated RTKs can recruit many downstream effector molecules. These molecules contain SH2 or PTB domains which bind phosphotyrosine residues on RTKs [29]. These proteins can either interact directly with the activated RTKs or they may interact with other docking proteins which are tyrosine phosphorylated by RTKs. Some of the well-known docking proteins which orchestrate the formation of large protein complexes downstream of RTK activation are FGF receptor substrate 2 (FRS2), insulin receptor substrate 1 (IRS1) and Grb2-associated binder 1 (Gab1). Some of the docking proteins have specificity in terms of which classes of RTKs they bind whereas other docking proteins bind RTK members across different families. A single RTK can bind different ligands. EGFR binds seven different ligands [30]. The strength of interaction with RTK varies for these different ligand molecules. The attributes of the active conformation of dimerized receptor differ greatly for different ligands. Different active dimer conformations of RTK activate different downstream signaling cascades [31]. Gene rearrangements and mutations confer certain structural features to RTKs that result in ligand-independent receptor dimerization and activation. Aberrant activation of RTKs by such means can lead to different pathophysiology. Gene rearrangements can lead to an abnormal coiled coil and leucine zipper conformations of the extracellular domain that induce ligand-independent association of RTKs. Mutations resulting in cysteine residues in the extracellular domain also can induce permanent association of two RTK monomers [32]. Transmembrane domain mutations also can result in constitutive dimerization of RTKs leading to certain pathophysiologies [33]. Apart from the classification outlined above, RTKs have also been categorized based on the commonality of downstream signaling and expression pattern across tissues. Three such classes are (1) EGFR/FGFR1/c-Met, (2) IGF-1R/NTRK2 and (3) PDGFRβ [34].

### Breast cancer stem cells and drug resistance

Despite the advent of new therapeutic avenues, tumor relapse remains to be a greater challenge in breast cancer management. There are various reasons for tumor recurrence including breast cancer stem-like cells (BCSCs) residing at primary tumor as well as at metastatic sites. CSCs are subpopulation of tumor cells which have the potential to self-renew and drive tumorigenesis. BCSCs are characterized by the expression of specific cell surface markers including EpCAM^+^/CD24^-^/CD44^+^ [35]. Moreover, it has been reported that CSCs also express high level of aldehyde dehydrogenase (ALDH) and it is associated with poor clinical outcome [36]. However, a recent study suggests that EpCAM^+^/CD24^-^/CD44^+^ CSCs are anatomically distinct from ALDH+ve CSCs. Molecular profiling of EpCAM^+^/CD24^-^/ CD44^+^ and ALDH+ve CSCs revealed that the former sub-populations exhibit quiescent, epithelial to mesenchymal transition (EMT) phenotype whereas ALDH+ve CSCs show epithelial phenotype with self-renewal capacity [37]. Tumor microenvironment consists of cancer-associated fibroblasts (CAFs), tumor-associated macrophages (TAMs), mesenchymal stem cells (MSCs) and other immune and vascular cells and involved in the maintenance of CSCs in breast cancer [11, 38]. RTK signaling in tumor and stromal cells plays a critical role in the regulation of both CD24^-^ and CD44^+^ and ALDH+ve CSC phenotypes. CSCs exhibit major impact on cancer therapy as they show resistance to conventional chemo therapies by expressing multi-drug resistance (MDR) genes. The CD44^+^/CD24^-^ tumor cell fraction is increased in breast cancer patients upon administration of neoadjuvant chemotherapy [39]. Moreover, paclitaxel and epirubicin-based chemotherapy is associated with enrichment of ALDH+ve cells in breast tumors [40]. Altered expression/dysregulation of RTKs is associated with BCSC phenotype and drug resistance. Several reports suggest the treatment of breast cancer with RTK-based therapies reverses the multidrug resistance [41–43]. The role of RTK signaling in regulation of CSC phenotype and drug resistance has been discussed further.

### Role of receptor tyrosine kinase (RTK) signaling in breast cancer progression

#### EGFR: A key regulator of cancer stem cell phenotype and metastasis in inflammatory breast cancer

**Fig. 2:**
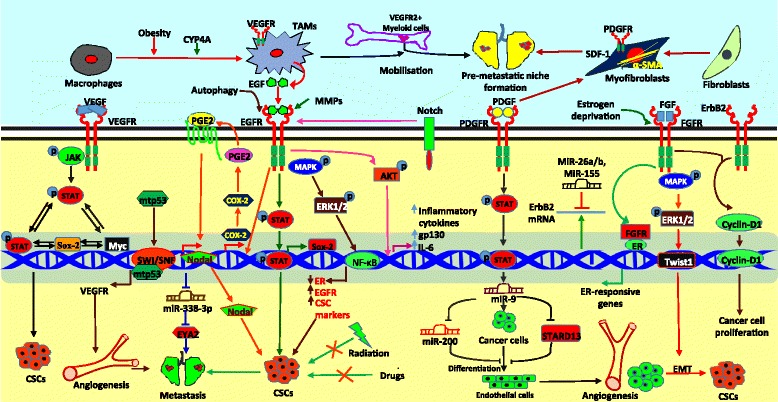
RTK-regulated signaling in breast cancer progression. VEGFR activates JAK/STAT signaling pathway to induce cancer stem cell phenotype through Myc and Sox2 expression. Mutant p53 induces the expression of VEGFR through the interaction with SWI/SNF complex. EGFR-regulated signaling also plays pivotal role in angiogenesis and metastasis. EGFR regulates the activation of JAK/STAT and MAPK signaling pathway to induce expression of Sox2 and other stem cell markers leading to enrichment of cancer stem cells. EGFR induces Akt phosphorylation to promote inflammation. PDGFR is expressed on stromal cells such as fibroblasts and is a marker of fibroblast activation. PDGFR-regulated STAT activation is involved in regulation of miR-9-mediated differentiation of cancer cells to endothelial cells leading to angiogenesis. FGFR-activated MAPK pathway induces EMT and CSC phenotype. Cooperation between the FGFR and HER2 regulates nuclear translocation of Cyclin D1 leading to enhanced cancer cell proliferation

EGFR is overexpressed in breast cancer tissues and is associated with higher aggressiveness and poor clinical outcomes [44, 45]. EGFR is a classic RTK and it undergoes homo or heterodimerization and trans-autophosphorylation upon ligand binding. EGFRs possess seven different cognate ligands including EGF, TGFα, betacellulin (BTC), heparin-binding EGF, amphiregulin (AREG), epiregulin, and epigen. The EGFR family consists of EGFR1 (EGFR, HER1, c-erbB1), HER2 (EGFR2, c-erbB2), EGFR3 (c-erbB3, HER3) and EGFR4 (c-erbB4, HER4) [46, 47]. Witton et al. have examined the expression of EGFR1, HER2, EGFR3 and EGFR4 using immunohistochemistry in 220 breast cancer patients and found overexpression of EGFR1 in 16.4%, HER2 in 22.8%, EGFR3 in 17.5%, and EGFR4 in 11.9% of breast cancer tissues. Increased expressions of EGFR1, HER2 or EGFR3 were associated with reduced survival whereas elevated level of EGFR4 was connected with better survival of breast cancer patients. It has been also reported that increased expressions of EGFR1, HER2 and EGFR3 were coupled with reduced expression of estrogen receptor (ER) [48]. Upon binding to the ligand, EGFR activates various downstream signaling molecules including Ras, PI3K, phospholipase C-γ (PLC-γ), and JAK leading to cell survival, cell growth, and tumor progression (Fig. 2) [6, 49, 50]. Various studies found that ER expression is inversely correlated with EGFR or cancer stem cell phenotype and that is well supported by the data that indicate higher expression of EGFR and presence of stem cell population in TNBCs which lack ER expression [51]. To investigate whether EGFR regulates stemness in breast cancer, Wise et al. have studied the enrichment of cancer stem cells under EGFR activation. They found that metalloproteinase-dependent activation of EGFR enriches CD44^+^/CD24^-^ stem cells in TNBC through the MAPK/ERK pathway (Fig. 2) [6]. Inflammatory breast cancer (IBC) (especially inflammatory TNBC) is a more lethal and aggressive form of breast cancer characterized by enrichment of chemo- and radio-resistant CSCs [52, 53]. Various reports suggest that EGFR signaling is important for IBC pathogenesis and progression [54, 55]. Activation of NF-κB in IBC leads to ER downregulation and EGFR and/or ErbB2 overexpression and MAPK hyper-activation. MAPK signature distinguishes IBC from non-IBC tumors better than ER-based stratification (54). Wang et al. have identified that EGFR/cyclooxygenase-2 (COX-2) axis-regulated nodal signaling promotes CSC phenotype and increases invasiveness of IBC cells through induction of EMT (Fig. 2) [55]. TGF-β-elicited EMT program augments expression of RTKs such as EGFR and IGF-1R which form cytoplasmic complexes with ER-α and Src leading to anti-estrogen resistance in breast cancer [56]. Syndecan-1 (CD138) is overexpressed and associated with cell proliferation and invasion, and emerged as an important drug target in IBC. Ibrahim et al. have established the relation between Syndecan-1 and EGFR in the regulation of cancer stem cell phenotype in inflammatory TNBC. Their studies revealed that Syndecan-1 regulates EGFR expression through activation of Notch signaling. Syndecan-1/Notch/EGFR crosstalk modulates interleukin-6 (IL-6), gp130 and other inflammatory cytokine expressions thereby promotes colony formation and stem cell marker expression through Akt-mediated NFκB activation (Fig. 2) [9].

Autophagy exhibits double-edged role in tumor progression depending on the context of a tumor. A recent study has revealed that autophagy regulates enrichment of ALDH+ve cancer stem-like cells via EGFR/Stat3 signaling in PyMT murine mammary cancer (Fig. 2) [57]. Tumor stroma also induces cancer stem cell phenotype by interacting with EGFR that is present on cancer cells through different downstream molecular players [58]. In the similar line of evidence, Yang et al. have reported that activation of EGFRs in cancer cells by TAMs leads to the Stat3-mediated Sox2 expression that resulted in increased cancer stem cell population and metastasis in murine breast cancer models (Fig. 2) [59].

#### VEGFRs: Master nodes in VEGF-regulated metastasis, tumor angiogenesis and lymphangiogenesis

Various studies established that angiogenesis is indispensable for breast tumor progression. VEGFs are potent proangiogenic factors that bind to three different types of VEGFRs, VEGFR1 (Flt1), VEGFR2 (KDR or murine homolog, Flk1). VEGFRs are expressed on cancer, endothelial and other stromal cells. VEGFRs are typical RTKs contain an extracellular domain for ligand binding, a transmembrane domain, and a cytoplasmic domain which includes a tyrosine kinase domain (TKD) [38]. VEGF-A binds to both VEGFR1 and VEGFR2 to induce tumor angiogenesis whereas VEGF-C and D interact with VEGFR3 to promote lymphangiogenesis in different types of cancer [38, 60]. However, Laakkonen et al. have reported that VEGF-C and VEGF-D-regulated VEGFR3 signaling induces tumor angiogenesis [61]. Chakraborty et al. have shown that osteopontin (OPN) augments VEGF-A expression in breast cancer cells and induces tumor growth and angiogenesis by regulating autocrine, paracrine and juxtacrine VEGF/VEGFR signaling in cancer and endothelial cells [62]. Srabovic et al. have reported that expression of VEGFR1 is significantly increased in breast tumor tissues as compared to benign tumors or healthy surrounding tissues, irrespective of the status of lymph node metastasis [63]. Kosaka et al. have identified elevated levels of VEGFR1 mRNA in peripheral blood of breast cancer patients and that is associated with cancer metastasis and recurrence and might be used for prognosis of breast cancer with basal-like and luminal type diseases [64]. In a recent study, Kapahi et al. have revealed that VEGFR1−710C/T polymorphism is associated with higher risk of breast cancer in North Indian population [65]. Ning et al. have revealed that VEGFR1 activation induces EMT of cancer cells thus promoting invasion and metastasis in breast cancer models [66]. Accumulated evidence suggests that infiltrated macrophages in tumor microenvironment promote malignant progression and enhance metastasis [11, 67]. A recent report has suggested that VEGFR1 signaling regulates obesity-induced tumorigenesis. Ablation of VEGF1 in obese animals reduced breast cancer growth and lung metastasis by decreasing M2 macrophage polarization and affecting glucose metabolism (Fig. 2) [67]. A recent evidence suggests that Flt1+ve metastasis-associated macrophages (MAMs), a subset of TAMs are enriched in metastatic breast cancer as compared to primary tumors. Flt1 signaling in MAMs regulates a set of inflammatory genes imperative for cancer cell survival after metastatic seeding. In addition, circulating VEGFR1+ve myeloid cells are involved in pre-metastatic niche formation [8, 68]. CYP4A polarized TAMs stimulate pre-metastatic niche formation and metastasis in lungs by mobilizing and recruiting VEGFR1+ve myeloid cells (Fig. 2) [68]. VEGR-2 is a key regulator of angiogenesis and overexpressed in breast cancer tissues [69]. Pfister et al. have studied the activation of VEGFR2 gene expression by mutant p53 in triple-negative breast cancer. In this study, they have shown that mutant p53 interacts with SWI/SNF and recruits to the promoter of VEGFR2 where this complex remodels the VEGFR2 promoter and induces the transcription leading to VEGFR-mediated breast tumor progression. These results indicate that mutant p53 gain of function is mediated by activation of VEGFR2 expression (Fig. 2) [70]. Collective evidences suggest that VEGFR2 exhibits prominent role in metastasis of breast cancer. However, the role of VEGFR2 in cancer cell invasion and migration is context-dependent. In breast tumor microenvironment, hypoxia induces c-Met/β1 integrin complex formation that results in higher invasion and migration potential of cancer cells. However, VEGF-activated VEGFR2 binds directly with c-Met and β1 integrin to prevent complex formation thus leading to sequestration of c-Met and β1 integrin [71]. Zhao *et al*. have found that VEGF drives VEGFR2 expression and subsequently activates JAK2/STAT3 signaling-mediated Myc and Sox2 expression. VEGF/VEGFR2 axis-established autocrine loop consisting of STAT3, Myc and Sox2 which implicated in enhancement of cancer stem-like cell phenotype in TNBC (Fig. 2) [10]. Nonetheless*,* CSCs are responsible for cancer cell metastasis, drug resistance, and tumor relapse, perturbing VEGFR2/STAT3/Myc/Sox2 axis might be useful in overcoming the chemo-resistance in triple-negative breast cancer.

Lymphangiogenesis, formation of new lymphatic vessel plays a major role in cancer cell dissemination and distant metastasis. Hence, lymphangiogenesis is proved to be a promising target for the treatment of breast cancer. However, unavailability of specific markers for studying lymphatic vessels and lymphogenic metastasis delays the development of anti-lymphangiogenic therapy for management of different types of cancer [72]. VEGFR3 is a RTK expressed on lymphatic endothelial cells (LECs) and it plays a key role in lymphangiogenesis [20]. A recent study suggested that CCL21/CCR7 chemokine axis expressed on breast cancer cells interacts with VEGFR3 present on LECs to induce tumor-dependant lymphatic vascular recruitment and thereby lymphangiogenesis in breast cancer [73]. Lymphangiogenesis is also imperative for metastasis in postpartum breast cancer. Recent reports suggest that COX-2 induces VEGFR3 expression and lymphangiogenesis via VEGF-C/VEGFR3 axis to promote nodal metastasis of postpartum breast cancer [74, 75]. VEGFR3 is indispensable for galectin-8-mediated-crosstalk involving the VEGF-C, podoplanin and integrin pathways leading to lymphangiogenesis in breast cancer [76]. Based on above findings, targeting lymphangiogenesis using anti-VEGFR3 therapy might be useful in preventing tumor cell metastasis and increasing survival of breast cancer patients.

#### PDGFR: promising role in tumor-stroma interaction in breast carcinoma

PDGFRs are type III RTKs that are highly expressed in breast tumor and stromal cells. The PDGFR family consists of PDGFR-α and β and both show similar kind of functions. PDGFR-α and β are structurally similar and contain extracellular domain which consists of five immunoglobulin (Ig) - like folds and intracellular domains that exhibit kinase activity and consists of 100 amino acid residues dissimilar to other RTKs. PDGFs mostly bind to Ig-like domains 2 and 3, and induce homo or heterodimerization of the receptors. Moreover, these receptors are further stabilized by direct receptor-receptor interactions through Ig-like domain 4 after dimerization [77]. Aberrant activity of PDGFRs in different types of cancer including breast drives tumorigenesis. Various studies reported that PDGFR expression is associated with poor prognosis of breast cancer patients and it has prognostic and predictive potentials [78–80]. PDGFR is known to regulate various downstream signaling networks including Stat3 to support breast tumor initiation and progression [72]. Park et al. have reported that AF1q-induced STAT3 activation enhances breast cancer cell proliferation, angiogenesis and metastasis through PDGFR/Src signaling cascade [7]. Apart from directly regulating cancer cells, PDGFRs are also found to be expressed in reactive desmoplastic stroma that shows its possible role in tumor-stroma interaction. Bhardwaj et al. have found that PDGFR is expressed by α-SMA-positive myofibroblasts (cancer associated fibroblasts, CAFs) and endothelial cells in the periepithelial stroma of breast cancer tissues (Fig. 2) [79]. Paulsson et al. have examined the prognostic role of stromal PDGFR-β expression using tissue microarrays (TMAs) of breast cancer. Their findings suggested that stromal PDGFR-β exhibits most prominent prognostic significance in the subset of breast tumors. They also found that enhanced PDGFR expression is associated with reduced ER and PR and higher HER2 expression as well as inceased proliferation rate and tumor size [80]. In a similar line of evidence, Pinto et al. have shown that malignant stroma induces luminal breast cancer cell proliferation and angiogenesis in estrogen free-conditions through the PDGFR signaling cascade [81]. These results indicate the major role of PDGFR in breast cancer progression in absence of ER signaling. This notion is further supported by the fact that PDGFR induces endothelial differentiation of TNBC cells using *in vitro* tube formation and *in vivo* xenograft models. Moreover, D'Ippolito et al. have delineated the molecular mechanism by which PDGFR-regulates endothelial differentiation of tumor cells in TNBC. PDGFR induced miR-9 expression promotes vasculogenic properties by targeting STARD13 and downregulating miR-200 in TNBC (Fig. 2) [13]. These results indicate that targeting PDGF/PDGFR in tumor microenvironment might be the promising therapeutic approaches for the treatment of TNBC.

#### FGFR: aberrantly expressed in breast cancer and implications in targeted therapy

The FGFR family members (FGFR1, FGFR2, FGFR3 and FGFR4) are comprised of an extracellular ligand-binding domain, a transmembrane domain and an intracellular tyrosine kinase (TK) domain. The extracellular domain has three Ig-like domains (IgI-III). The FGFs binding to FGFR leads to dimerization and subsequent activation of the intracellular kinase domain resulting in cross-phosphorylation of tyrosine residues present on the cytoplasmic tail of the receptor [82]. Ras/MAPK and PI3K/Akt pathways are activated downstream to these receptors upon ligand stimulation. These pathways are known to be aberrantly activated in breast cancer and are involved in cell survival, proliferation, apoptosis and migration [83, 84]. The FGFRs harbour genetic aberrations such as amplifications of FGFR1, FGFR2 and FGFR4 and mutations in FGFR2 and FGFR4 genes in breast cancer [84–87]. Metastatic lobular breast carcinoma which shows poor response to chemotherapy demonstrates amplification of FGFR1 gene with implications in targeted therapy [86]. Formisano et al. have demonstrated that ER+ breast cancer shows amplification of FGFR1. They found that FGFR associates with ERα in nuclei of breast cancer cells and regulates ER-dependent genes in the presence of estrogen deprivation. In addition to ER+ breast cancer, amplification of FGFR1 gene correlated with poor prognosis in HER2- breast cancer [88]. Moreover, elevation of FGFR regulates tumor stroma remodelling and tumor recurrence in FGFR1-driven breast cancer [2]. Hence, studies with combinational therapies, targeting FGFR1 and other RTKs showed better results in cancer treatment as compared to targeting a single RTK. Single nucleotide polymorphisms (SNPs) in FGFR2 have been associated with an increased risk of ER+ and PR+ breast cancer [89]. Cerliani et al. have observed the interaction of FGFR2 with progesterone and STAT5 in breast tumor resulted in increased transcription of PR/STAT5-regulated genes [90]. Association of FGFR2 and FGFR3 expression with ER+ breast cancer progression was observed [91]. Even though, role of FGFR3 in breast cancer progression has not been studied well, splice variants of FGFR3 are known to localize to nucleus of breast epithelial cancer cells [92]. Koziczak et al. have shown that FGFR4 and ErbB2 co-operately regulate cyclin D1 expression to promote cell proliferation in breast cancer [93]. FGFR signaling-regulated ERK1/2- mediated Twist1 positive feedback loop stabilizes a CD44^high^ drug-resistant phenotype following ErbB inhibition (Fig. 2) [94]. Based on above findings, it is clear that FGFRs are mechanistically linked to the functions of other RTKs and drug resistance and may be a potential targets for treatment of breast cancer.

### Role of miRNAs and lncRNAs in regulation of RTK signaling

In recent years, several studies have reported the role of microRNAs (miRNAs) and long non-coding RNAs (lncRNAs) in regulating the expression of components of different RTK signaling pathways. Tan et al. have shown that the level of ErbB2 in tamoxifen-resistant ER^+^ breast cancer is tightly regulated by interplay between miR-26a/b and human antigen R (HuR) (Fig. 2) [95]. miR-34a and miR-155 also regulate expression of ErbB2 at the post-transcriptional level (Fig. 2) [96, 97]. miR-24 targets two regulators (tyrosine-protein phosphatase non-receptor type 9 (PTPN9) and receptor type tyrosine protein phosphatase F (PTPRF)) of EGFR activation, thereby promoting metastasis of breast cancer [98]. EGFR is a direct target of miR-206 in breast cancer and the latter is induced in nuclear factor (erythroid-derived 2)-like 2 (NRF2)-deficient breast cancer [99]. In human breast cancer, H19 lncRNA-derived miR675 targets c-Cbl and Cbl-b, E3 ubiquitin ligases which are known to degrade EGFR and c-MET thereby increases the stability of latter [100]. lncRNA CYTOR regulates the breast cancer progression through EGFR dependent pathway [101]. Another lncRNA, BCAR4 enhances the activity of ErbB2/3 receptors [102]. Role of different miRNAs and lnRNAs in the regulation of RTK signaling components are listed in Table 1.

**Table 1:** Role of miRNAs and lncRNAs in the regulation of RTK signaling

Serial No.	Molecule	miRNA/lncRNA	Target RTK pathway	Pathological function
1	MiR-26a/b	miRNA	EGFR (ErbB2)	Regulates expression of ErbB2; competes with HuR for binding to its 3’UTR in tamoxifen-resistant ER^+^ breast cancer [95]
2	MiR-34a	miRNA	EGFR (ErbB2)	Downregulates expression of ErbB2 [96]
3	MiR-155	miRNA	EGFR (ErbB2)	Downregulates HDAC2, a transcriptional activator of ErbB2; binds directly to a regulatory sequence over the coding region of ErbB2 [97]
4	MiR-24	miRNA	EGFR	Regulates levels of phospho-EGFR by targeting phosphatases, PTPN9 and PTPRF [98]
5	MiR206	miRNA	EGFR, MET	Downregulates EGFR and c-MET [99]
6	H19/miR675	lncRNA/daughter miRNA	EGFR, MET	Stabilizes EGFR and c-MET by targeting c-Cbl and Cbl-b [100]
7	CYTOR	lncRNA	EGFR	Regulates expression of EGFR pathway specific genes [101]
8	BCAR4	lncRNA	ErbB2/3	BCAR4 enhances ErbB2/3 activity in tamoxifen-resistant breast cancer [102]

### Role of RTK signaling in drug resistance

**Fig. 3:**
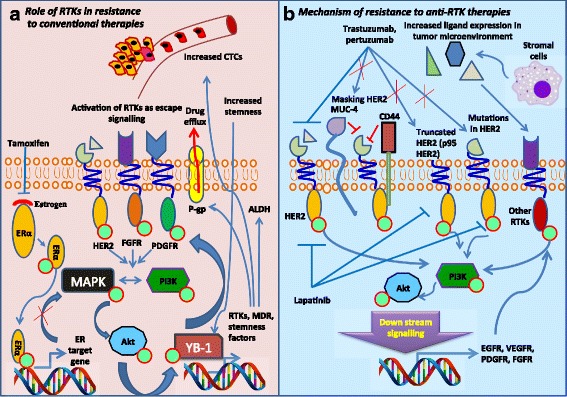
RTK signaling in drug resistance. **a** Conventional chemotherapeutic agents reduce the cancer progression through the inhibition of MAPK/PI3K/Akt signaling axis. Amplification and overexpression of RTKs including EGFR, HER2 and PDGFR reinforce the activation of PI3K/Akt/YB-1/RTK axis to maintain drug resistance; increases the kinase activity and thereby leading to cancer progression, drug efflux and cancer stemness. **b** Cancer cells exhibit resistance to RTK therapy due to disruption of interaction between drug and receptor or activation of alternate RTK signaling

Endocrine therapy is the treatment that specifically blocks the function of ER signaling using antagonists (tamoxifen, fulvestrant) or estrogen deprivation [103]. Almost 20% of the patients acquire resistance to ER-targeted therapy via activation of escape signaling pathways to overcome estrogen dependency [104]. Overexpression or activation of RTKs such as EGFR, HER2 and IGF1R leads to downregulation of ER and resistance to tamoxifen through activation of PI3K/Akt and MAPK pathways (Fig. 3) [105, 106]. EGFR/MAPK axis promotes phosphorylation of AF-1 domain of ER to enhance the ligand-independent activation of ER signaling [106, 107]. Activation of EGFR/ErbB2 signaling in tamoxifen-resistant ER+ breast cancer cells induces highly aggressive stem cell phenotype in these cells [108–110]. Inhibition of EGFR signaling using erlotinib considerably reduces the cancer stemness and reverses the endocrine resistance by inducing the expression of ER [111]. Moreover, HER2 amplification in ER-resistant breast cancer correlates with the ALDH+ stem cell population [108]. CSC population expresses a very high level of HER2 mRNA and protein as compared to the non-CSC population in endocrine-resistant patients. Higher activation of EGFR/HER2 might be the driving force in enriching CSC population in tamoxifen-resistant breast cancer [36, 108]. Association of HER2 expression with ER resistance has been explained in several reports. Whole exome sequencing studies revealed 13 mutations in different domains of HER2 in ER+ endocrine-resistant metastatic breast cancer patients [112]. These mutations produce different level of resistance to tamoxifen and fulvestrant in ER+ breast cancer cell lines. Moreover, ER cofactors, HOXB3 and HOXB7 are found to be overexpressed in tamoxifen-resistant breast cancer cells and enhance CSC phenotype. Myc-mediated transcriptional repression of miR-375 and miR-196a enhances the expression of HOXB3 and HOXB7 respectively [113, 114]. Retinoblastoma binding protein 2 (RBP2), an ER co-regulator is overexpressed in tamoxifen-resistant breast cancer patients and increases the stability of RTKs such as EGFR and HER2. Moreover, RBP2-ER-NRIP1-HDAC1 complex activates IGF1R through transcriptional repression of IGFBP4 and 5 [115]. Another ER transcriptional coactivator, mediator subunit 1 (MED1) is overexpressed in circulating tumor cells and primary breast tumor tissues following tamoxifen treatment leading to HER2-mediated ER resistance. HER2-mediated phosphorylation of MED1 recruits the transcriptional corepressors such as HDAC1, N-CoR and SMART to the promoter of the ER-regulated genes in HER+ tamoxifen-resistant cells [116, 117].

Apart from the endocrine therapy, other types of treatment such as surgery, radiation therapy and cytotoxic drugs are also available for breast cancer. Mainly, anthracyclines (DNA damaging agents) and taxanes (microtubule-stabilizing agents) are widely used for breast cancer as adjuvant or neoadjuvant therapies [118]. However, the resistance to cytotoxic cancer drugs is the major drawback in cancer treatment. Multidrug resistance is mainly associated with cancer stemness and drug efflux driven by various survival signals [119]. Importantly, RTKs are key regulators of cancer stemness and associated with drug resistance in breast cancer cells. In general, various RTKs activate PI3K/Akt signaling to induce the expression of cancer stemness factors, multidrug resistance associated proteins and membrane transporters in cancer cells. Accumulating evidence clearly suggest that upregulation of RTKs including EGFR, HER2, VEGFR and IGF-1R in course of chemotherapy is associated with overexpression/activation of drug efflux transporters [41, 42]. Jin et al. have shown the strong positive correlation between p-glycoprotein expression and EGFR with overall and disease-free survival [43]. Moreover, higher expressions of EGFR and HER2 are detected in doxorubicin-resistant MCF7 cells as compared to the doxorubicin-sensitive MCF7 cells. Overexpression of HER2 also induces resistance to various chemotherapeutic agents such as taxane, cyclophosphamide, methotrexate, epirubicin in breast cancer [120]. Moreover, HER2 expressing circulating tumor cells (CTCs) shows less sensitivity to the various chemotherapeutic agents including doxorubicin, docetaxel and 5-fluorouracil as compared to HER-negative CTCs [121]. Overexpression of RTKs is correlated with expression of transcription factors linked to drug resistance in breast cancer. YB-1 is a transcriptional/translational regulator and overexpressed in cancer stem cells. Nuclear localization of YB-1 is reported in cancer relapse and drug-resistant patients irrespective of ER and HER2 status. RTK-regulated PI3K/Akt phosphorylates YB-1 at Ser-102 to facilitate the nuclear localization. Furthermore, nuclear YB-1 binds to the specific promoter region and transcriptionally activates the expression of RTKs including EGFR, HER2 and VEGFR. Disturbance in YB-1/RTKs self-reinforcing loop significantly reduces the cancer stemness and drug efflux in breast cancer cells [122]. Moreover, YB-1 transcriptionally increases the expression of p-glycoproteins (MDR-1 and MDR-3) provokes the multidrug resistance in breast cancer (Fig. 3) [123, 124]. TAMs are known to influence the maintenance of suitable microenvironment for cancer stem cells and sustained drug resistance in breast cancer. TAMs produce the higher level of cytokines, TGFα, EGF, FGF and VEGF in the tumor microenvironment. Higher levels of these ligands activate RTK signaling in breast cancer as well as macrophages [125]. A strong correlation between EGFR expression and CD163+ macrophages were found in tamoxifen-resistant breast cancer patients [126]. Moreover, TAMs upregulate the cancer stemness associated genes along with increased drug efflux and chemoresistance in preclinical breast cancer model [127].

### Receptor tyrosine kinase (RTK)-targeted cancer therapeutics

Breast cancer is a heterogeneous disease which has been characterized molecularly into five subtypes depending on expression of ER, PR and HER2. These subtypes consist of Luminal A (low grade, ER+/PR+, HER2-, low Ki67), Luminal B (ER+/PR+, HER2+ or HER2-, high Ki67), TNBC or basal-like (ER-/PR- and HER2-), HER2-enriched and normal-like breast cancer [128]. For hormone receptor-positive breast cancer (luminal A and B), hormone therapy consists of selective estrogen receptor modulators (tamoxifen and raloxifene) is routinely used as adjuvant therapy [129]. Since TNBC or basal-like and HER-enriched breast cancer do not express hormone receptors so that hormone therapy is not effective in these subtypes. However, due to the prominent expression of RTKs in TNBC and HER2-enriched subtypes, blocking the functions of RTKs is one of the promising approaches for management of TNBC and HER2-enriched breast cancer. So far, various strategies have been adopted for inhibition of RTK-dependent signaling. Mutations or overexpression of EGFR genes leads to tumor progression and drug resistance in various cancer types including breast [127]. Therefore, EGFR holds the potential to be an attractive drug target in breast cancer, and the EGFR inhibitors, including small molecule inhibitors and monoclonal antibodies (mAbs), have been developed and some are currently used in clinics. Overexpression of HER2 is frequently found in breast cancer. Several HER2-targeting drugs were developed and are currently used for the treatment of breast cancer.

Trastuzumab (Herceptin) is a humanized mAb which targets the extracellular domain of HER2 in HER2+ breast cancer and it has been reported to enhance survival of patients at early and late stages of breast cancer [130]. However, the exact mechanism through which trastuzumab exhibits its therapeutic effect is not well understood. De et al. have reported that trastuzumab inhibits HER2-HER3 heterodimerization which is known to occur in a ligand-independent manner in HER2+ breast cancer. Several reports also suggested that trastuzumab might induce HER2 degradation but the underlying mechanism is unexplored [131]. Although treatment with trastuzumab significantly improves disease outcome, resistance to trastuzumab is a major barrier to treat HER2-positive breast cancer. Approximately 65 % of HER2-positive breast cancer patients do not respond to primary trastuzumab treatment. Moreover, a majority of patients those who originally respond well to trastuzumab therapy show tumor relapse later [132, 133]. In 2013, FDA approved an antibody-drug conjugate T-DM1 or trastuzumab emtansine or ado trastuzumab emtansine (trade name Kadcyla) for the treatment of HER-positive metastatic breast cancer patients who has been previously treated with trastuzumab and a taxane. T-DM1 consists of trastuzumab and cytotoxic agent emtansine (DM1) which kills the cancer cells by binding to tubulin [134]. A random trial on 991patients with HER2-positive advanced breast cancer showed higher median progression-free survival in T-DM1-treated patients compared to lapatinib plus capecitabine-treated ones [135]. However, a recently completed phase III trial using trastuzumab plus taxane, T-DM1 plus placebo, T-DM1, or T-DM1 plus pertuzumab regimens at standard doses in 1095 HER2-positive advanced breast cancer patients. No significant increase in progression-free survival in T-DM1 and T-DM1 plus pertuzumab groups was observed as compared with trastuzumab plus taxane; although, T-DM1 containing arms showed better tolerability [136]. Pertuzumab (trade name perjeta) is another monoclonal antibody against HER2 which has been approved for neo-adjuvant or adjuvant therapy of HER2-positive advanced breast cancer in a combination with trastuzumab and docetaxel. Clinical trials have demonstrated that breast cancer patient’s administered with combination of pertuzumab, trastuzumab and docetaxel had enhanced progression-free survival compared to control group [137, 138].

TNBC or basal-like breast cancer is known to be negative for HER2, shown to express EGFR in 40% of the patients, of those 18% of patients are reported to have amplified EGFR gene. Hence, EGFR is one of the important targets for HER2 negative breast cancer including TNBCs. Lapatinib (Tykerb), a dual tyrosine kinase inhibitor, binds to ATP binding pocket of EGFR and HER2 kinase domain and blocks ATP binding thereby leading to inhibition of EGFR and HER2 kinase activity. The tyrosine kinase inhibitors (TKIs) are known to be used as an alternate therapeutic regimen in HER2+ breast cancer patients with trastuzumab resistance [139, 140]. Moreover, lapatinib has been used in combination with other anticancer drugs, capecitabine or letrozole. These combination therapies showed higher disease-free survival in HER2+ metastatic breast cancer patients [141, 142]. Multiple clinical trials have been conducted to assess the efficacy and toxicity of TKIs either alone or in combination with other drugs in breast cancer. Unfortunately, the outcomes of these trials have been disappointed so far. Few trials and their outcomes are enlisted in Table 2. Phase II clinical trials of gefitinib or erlotinib have shown poor overall response rate (ORR) while clinical trial with gefitinib in combination with epirubicin and cyclophosphamide showed no significant difference in pathologic complete response in ER-negative breast cancer [142–146]. Further, afatinib, a second-generation irreversible EGFR TKI, has shown no objective responses in phase II trial in metastatic TNBC patients [147].

**Table 2:** Current anti-RTK therapy

Clinical studies of RTK-targeted therapeutics in breast cancer				
Molecule		Target	Outcome	Ref.
Gefitinib +Epirubicin and Cyclophosphamide		EGFR	No significance	[146]
Cetuximab + Carboplatin		EGFR	Overall response rate: 6% (Carb), 16% (Carb + cetux), TTP - 2.1 month	[148]
Cetuximab + Cisplatin		EGFR	Overall response rate: 10% (cis), 20% (cis + cetux) P=0.032	[149]
Cetuximab + Ixabepilone		EGFR	No significance	[150]
Cetuximab + Irinotecan		EGFR	Overall response rate: 11%	[151]
Panitumumab + Epirubicin, Fluorouracil and Cyclophosphamide (EFC) + Docetaxal		EGFR	Pathological complete response: 47%	[152]
Cetuximab + Docetaxal		EGFR	Pathological complete response: 24%	[153]
Panitumumab+Paclitaxal and Carboplatin		EGFR	Overall response rate: 46%	[154]
Erlotinib + Bendamustine		EGFR	Cause excessive toxicity with severe, prolonged lymphopenia	[155]
Paclitaxal + Bevacizumab		VEGFR	Higher progression free survival	[156]
Bevacizumab + Capacitabine		VEGFR	Higher progression free survival	[157]
Sunitinib + Docetaxal		VEGFR, PDGFR	No significant difference in progression free survival	[158]
Currently investigated clinical trials of targeting RTK in breast cancer				
Molecule	Type	Target	Phase of study	Mechanism
Trastuzumab	Humanized MAb	HER2	In clinical use	Inhibits HFR2 and HER3 dimerization, induces ADCC [159]
Cetuximab	ChimaricMAb	EGFR	Phase I, II	Enhances sensitivity to DNA-damaging agents in *BRCA1*-mutated and *PTEN*-wild-type TNBC, Induces NK cell mediated ADCC [160, 161]
Panitumumab	Humanized MAb	EGFR	Phase II	Enhances sensitivity to DNA-damaging agents in *BRCA1*-mutated and *PTEN*-wild-type TNBC [161]
Nimotuzumab	Humanized MAb	EGFR	Phase I	Induces NK cell mediated ADCC [162]
Necitumumab	Humanized MAb	EGFR	Phase II	Inhibits downstream targets in EGFR pathway, induces ADCC [163]
Gefitinib	Reversible TKI	EGFR	Phase I, II	Reverses TAM resistance by up-regulating the ERα [164]
Erlotinib	Reversible TKI	EGFR	Phase I, II	Suppresses CDK2 activity [165]
Lapatinib	Reversible TKI	EGFR, HER2	In clinical use	Used as an alternate therapy in trastuzumab resistant HER2 positive breast cancer [139]
Afatinib	Irreversible TKI	EGFR, HER2	Phase II	Inhibits EGFR and HER2 signalling irreversibly [166]
Varlitinib	Reversible TKI	EGFR, HER2, ErbB4	Phase II	Inhbits HER/MAPK signalling in TNBC [167]
Dacomitinib	Irreversible TKI	EGFR, HER2, ErbB4	Phase 1, Solid tumors	Inhibits HER2, EGFR, HER4, Akt and ERK phosphorylation and show high antitumor effect in trastuzumab and lapatinib resistant HER2 overexpressing breast cancer [168]
Sapitinib	Reversible TKI	EGFR, HER2, ErbB3	Phase 1, Solid tumors	Showed higher inhibitory potential in tamoxifen resistant breast cancer [169]
Vandetanib	TKI	EGFR, VEGFR2-3, RET	Phase I, II	Targets angiogenesis by inhibiting VEGFR2 and 3 signalling along with EGFR pathway [170]
Neratinib	Irreversible TKI	EGFR, HER2, ErbB4	Phase I, II, III	Irreversibly blocks EGFR and HER2 pathway [171]
BMS-690514	Irreversible TKI	EGFR, HER2, ErbB4, VEGFR1-3	Phase 1, Solid tumors	Irreversibly blocks EGFR and HER2 pathway leading to inhibition of their downstream signaling pathways [172]
AEE788	Reversible TKI	EGFR, ErbB2, VEGFR	Phase I	Targets angiogenesis by inhibiting VEGFR2 and 3 signalling along with EGFR pathway [173]
Lucitanib	TKI	FGFR 1-2, VEGFR 1-3, PDGFRα/β	Phase II	Show anti-angiogenic and anti-tumoral activity by targeting FGFR and VEGFR [174]

There have been six clinical trials with anti-EGFR mAbs to explore their efficacy and safety in TNBC patients as given in Table 2. Carey et al. have performed a clinical trial in metastatic advanced recurrent breast cancer to examine the efficacy of cetuximab or cetuximab in combination with carboplatin. Cetuximab in combination with carboplatin demonstrated higher response rate as compared to carboplatin alone. However, 13 out of 18 treated patients showed active EGFR signaling that indicates cetuximab failed to inhibit the EGFR pathway [148]. Higher response rate in cisplatin-cetuximab treated patients (20%) as compared to cisplatin-treated group (10%) has been reported in advanced TNBC. However, the outcomes were not statistically significant [149]. Similarly, a phase II trial of ixabepilone alone and ixabepilone plus cetuximab in patients with advanced/metastatic TNBC was conducted by Tredan *et al.* This study has shown no improvement in response rate [150]. Meanwhile, irinotecan and cetuximab were shown increased response rate in TNBC patients compared to other subtypes, however, the results were not statistically significant [151]. Modest response was observed when operable TNBC patients were treated with standard FEC (5-fluorouracil, epidoxorubicin, and cyclophosphamide) following preoperative chemotherapy consisting of panitumumab or cetuximab combined with docetaxel [152, 153]. Higher CD8+ tumor infiltrating lymphocytes (TILs) were spotted in the tumor microenvironment in response to EGFR mAb neoadjuvant therapy. Overall, the outcome of clinical trials of EGFR mAbs in TNBC seems to be slightly better than that of EGFR TKIs. Several trials using anti-RTK therapy and their outcomes are enlisted in Table 2 [146, 154–174].

### Challenges in targeting RTKs in breast cancer: emphasis on compensatory elements

RTK-targeting therapeutic drugs are known to reduce multidrug resistance and CSC phenotype in breast cancer cells. However, cancer cells exhibit the resistance to RTK inhibitors in clinical and preclinical models. For example, HER2-targeted therapies (trastuzumab, pertuzumab, TDM1 and lapatinib) are known to impede primary tumor progression and cancer relapse but still drug resistance is observed in approximately 80% of HER2+ metastatic breast cancer patients [142]. Similarly, many cancer types including breast often acquire resistance to various RTK inhibitors such as VEGFR inhibitors (bevacizumab) [175], EGFR inhibitors (gefitinib) [176], FGFR inhibitors (AZD4547) [177]. Several mechanisms have been derived to describe the occurrence of resistance to RTK inhibitors. Several mutations in RTKs and their downstream targets and the activation of multiple other RTKs are the major compensatory elements instigated the survival pathways and resistance to anti-RTK therapies in breast cancer. IGF1R, EGFR, AXL, VEGFR are other RTK members share common downstream signaling molecules such as PI3K/Akt/mTOR and MAPK with HER2 in breast cancer [178]. Moreover, IGF1R overexpressed in HER2+ breast cancer and forms a heteromeric complex with HER2 and HER3 to activate PI3K signaling pathway. These heteromeric complex formation with HER family proteins have been associated with trastuzumab resistance in HER2+ metastatic breast cancer patients [179]. Combination of anti-HER2 drugs with anti-IGF1R mAbs (metformin and figitumumab) have reported to produce synergetic effects in breast cancer cells. C-Met is the RTK, frequently expressed in HER2+ breast cancer patients and contributes to trastuzumab resistance. Upregulation of c-Met protects the cancer cells from trastuzumab via abrogating p27-induction whereas inhibition of c-Met sensitizes the cancer cells to trastuzumab treatment [180]. c-Src-mediated phosphorylation of EGFR at Tyr845, Tyr992, and Tyr1086 is associated with resistance to anti-EGFR therapy in breast cancer. Activation of c-Met during EGFR treatment facilitates c-Src kinase-associated phosphorylation and cell growth in breast cancer cells. Furthermore, a combination of c-Met targeting small molecule inhibitors along with EGFR inhibitor decreases EGFR phosphorylation and kinase activity via inhibiting c-Src kinase thereby reduces the EGFR resistance [181]. Increased copy number of FGF3/4/19 has been reported in lapatinib and trastuzamab-resistant tumors. Higher expression and phosphorylation of FGFR is correlated with reduced disease-free survival and anti-HER2 therapy resistance in breast cancer patients. Activation of FGFR further stimulates the phosphorylation of non-receptor kinases such as MAPK and PI3K/Akt through the activation of phospholipase Cγ in tamoxifen-resistant breast cancer [182]. Amplifications and mutations in RTK dependent downstream target genes (PI3KCA or Akt) bypass the role of RTKs in their activation so that produce uninterrupted activation of growth signaling in breast cancer cells. Mutation in PI3CA is strongly associated with ErbB2-overexpression and lymph node metastasis [183].

Bevacizumab is the first anti-VEGFR drug approved by US FDA for the treatment of breast cancer but it is discontinued eventually due to the occurrence of resistance to it. Anti-VEGFR therapy induces hypoxia in the tumor microenvironment and its lead to increase in the aggressiveness of breast cancer. Under hypoxic stimuli, stromal cells secrete very high level of cytokines that activate alternate angiogenic pathways and increase the cancer stemness and autophagy [175]. Ephrin- A1 and B2 are proangiogenic factors, important for the remodeling and maturation of new blood vessels. Hypoxia mediates the upregulation of ephrin and the expression of ephrins is strongly associated with resistance to VEGFR therapy. Several proangiogenic factors such as angiopoietin 2 (ANG-2), EGF, bFGF, keratinocyte growth factor, IGF-1, TGF-β, TNF-α and interleukins (IL-1, IL-8, IL-12 and IL-17) have been implicated in hypoxia-associated tumor refractoriness to anti-VEGFR therapy [184]. Secretion of IL-17, G-CSF, IL-6 and SDF1in tumor microenvironment recruits CD11b+Gr1+ myeloid cells to tumor and conferring Bv8-associated VEGFR-independent angiogenesis leads to resistance to anti-VEGFR therapy. Depletion of CD11b+Gr1+ myeloid cell infiltration by Bv8 neutralizing antibodies sensitizes the cancer cells to VEGFR-targeted therapy [185].

Impaired interaction between anti-RTK agents and its respective receptor is another reason behind the development of resistance. This might be due to the higher existence of masking proteins in close proximity to the receptors, structural changes in the receptor and lack of expression of targeted domain. Mucin-4 and CD44 are the cell surface proteins overexpressed in trastuzumab resistant breast cancer patients. Expression of these proteins in close proximity to the HER2 epitope masks the interaction between trastuzumab and HER2 and increase the breast cancer growth [186, 187]. On other hand, expression of a truncated version of HER2 overrides trastuzumab sensitivity in breast cancer. p95^HER2^ forms heterodimer with HER3 protein and activates downstream signaling in a ligand-independent manner (Fig. 3) [188]. Eliyatkin *et al*. have shown that 28% of the patients who develop trastuzumab resistance have higher expression of p95^HER2^. However, low level of p95^HER2^ expression is found in trastuzumab-sensitive patients as well [189]. Moreover, mutations in HER2 could perturb the antibody recognition or physical interaction between drug and receptor. T798M mutation in HER2 showed increased autocatalytic activity and expression of EGFR ligands lead to 10-fold changes in IC50 of lapatinib in human breast cancer cells. Moreover, EGFR targeting antibody, cetuximab or lapatinib revert the trastuzumab resistance in these T798M specific cells [190]. Hanker et al. have shown that patients with HER2^L869R^ mutation acquire secondary mutation at HER2^T798I^ as subsequent response to neratinib treatment. Molecular modeling studies suggested that HER2^T798I^ has increased isoleucine content in its protein structure and that reduces the binding between neratinib and HER2 [191].

## Conclusions

Overexpression or dysregulation of RTKs in breast cancer cells leads to accelerated tumor growth, angiogenesis and metastasis through the activation of various downstream signaling pathways. RTKs play a key role in cancer stemness and drug resistance to various conventional anti-cancer therapies. Hence, targeting RTKs is one of the more promising approaches for the management of breast cancer. Many of RTK targeting drugs are in clinic for the treatment of breast cancer. However, resistance-driven by mutations in RTKs and alternate pathway activation limits the use of anti-RTK therapeutics for the treatment of metastatic breast cancer. The comprehensive mechanism underlying the resistance to anti-RTK therapy needs to be investigated to develop therapeutic regimens for successful treatment of anti-RTK therapy-resistant breast cancer.

## Abbreviations

CSCs: Cancer stem cells
EGFRs: Epidermal growth factor receptors
FGFRs: Fibroblast growth factor receptors
FRS2: FGF receptor substrate 2
Gab1: Grb2-associated binder 1
IGFRs: Insulin-like growth factor receptors
IRS1: Insulin receptor substrate 1
JAK: Janus kinase
MAPK: Mitogen-activated protein kinase
NGF: Nerve growth factor
PDGFRs: Platelet-derived growth factor receptors
PI3K: Phosphoinositide 3-kinase
PTB: Phosphotyrosine-binding
RTKs: Receptor tyrosine kinases
SCF: Stem cell factor
SH2: Src homology 2
STAT: Signal transducer and activator of transcription
TKD: Tyrosine kinase domain
VEGFRs: Vascular endothelial growth factor receptors
